# Barreras en la atención de la salud en niños con defectos congénitos atendidos mediante el programa AIVA

**DOI:** 10.15446/rsap.V25n3.107641

**Published:** 2023-05-01

**Authors:** Alba Ibáñez-Morantes, Karen Sarmiento-Acuña, Fernando Suárez-Obando, Ignacio Zarante

**Affiliations:** 1 AI: MD. Residente de Patología. Instituto de Genética Humana. Facultad de Medicina, Pontificia Universidad Javeriana. Bogotá, Colombia. aciml217@hotmail.com Pontificia Universidad Javeriana Instituto de Genética Humana Facultad de Medicina Pontificia Universidad Javeriana Bogotá Colombia; 2 KS: MD. M.SC. Toxicología. Esp. Gerencia en Salud y Salud en el Trabajo. Departamento de Ciencias Fisiológicas. Facultad de Medicina, Pontificia Universidad Javeriana. Bogotá, Colombia. ksarmiento@javeriana.edu.co Pontificia Universidad Javeriana Departamento de Ciencias Fisiológicas Facultad de Medicina Pontificia Universidad Javeriana Bogotá Colombia ksarmiento@javeriana.edu.co; 3 FS: MD. Esp. Genética. Ph.D. Genética. Instituto de Genética Humana. Facultad de Medicina, Pontificia Universidad Javeriana. Bogotá, Colombia. fernando.suarez@javeriana.edu.co Pontificia Universidad Javeriana Instituto de Genética Humana Facultad de Medicina Pontificia Universidad Javeriana Bogotá Colombia; 4 IZ: MD. M. Sc. Ciencias Biológicas. Ph.D. Genética. Instituto de Genética Humana. Hospital Universitario San Ignacio. Facultad de Medicina, Pontificia Universidad Javeriana. Bogotá, Colombia. izarante@javeriana.edu.co Pontificia Universidad Javeriana Hospital Universitario San Ignacio Facultad de Medicina Pontificia Universidad Javeriana Bogotá Colombia izarante@javeriana.edu.co

**Keywords:** Accesibilidad a los servicios de salud, niños con discapacidad, trastornos de la visión, pérdida auditiva, anomalías congénitas *(fuente: DeCS, BIREME)*, Access to health services, quality of life disabled children, loss of vision, hearing loss, congenital abnormalities *(source: MeSH, NLM)*

## Abstract

**Objetivo:**

Identificar las barreras en la atención de la salud en pacientes con defectos congénitos (DC) visuales y auditivos atendidos mediante el Programa de Atención Integral a Familias con Enfermedades Huérfanas con Compromiso Visual y/o Auditivo (AIVA), en Bogotá D. C., Colombia.

**Materiales y Métodos:**

Estudio transversal realizado en 58 niños con diagnóstico de DC con posible compromiso visual o auditivo. La población de estudio se seleccionó de la base de datos del programa AIVA, y para la obtención de los datos se les solicitó a los padres o representantes legales llevar a los niños a una valoración médica inicial y responder una entrevista. Las barreras se clasificaron según el modelo de cobertura efectiva de Tanahashi y los datos se analizaron mediante estadística descriptiva; se calcularon frecuencias absolutas y relativas para las variables cualitativas, y medias y desviaciones estándar o medianas y rangos intercuartílicos (según la distribución de los datos determinada con la prueba Shapiro-Wilk) para las cuantitativas.

**Resultados:**

El 81,03% de los padres o representantes legales manifestaron al menos una barrera, siendo las más frecuentes las de disponibilidad (49,38%), seguidas de las de accesibilidad (32,24%), aceptabilidad (11,83%) y contacto (6,53%).

**Conclusión:**

La mayoría de los padres o representantes legales entrevistados reportaron barreras de acceso a los servicios de salud. Dado que estas repercuten de forma negativa en la salud de los niños con DC, se requieren intervenciones conjuntas para reducirlas y así garantizar mejores condiciones de salud en los niños con DC y compromiso auditivo o visual.

## Abstract (en)

**Objective:**

To identify the barriers in health care in patients with visual and auditory congenital defects (CD) treated through the Comprehensive Care Program for Families with Orphan Diseases with Visual and/or Auditory Compromise (AIVA), in Bogotá D. C., Colombia.

**Materials and Methods:**

Cross-sectional study carried out in 58 children diagnosed with CD with possible visual or auditory compromise. The study population was selected from the AIVA program database, and to obtain the data, parents or legal representatives were asked to take the children for an initial medical evaluation and answer an interview. The barriers were classified according to Tanahashi's effective coverage model and the data were analyzed using descriptive statistics; absolute and relative frequencies were calculated for the qualitative variables, and means and standard deviations or medians and interquartile ranges (according to the distribution of the data determined with the Shapiro-Wilk test) for the quantitative variables.

**Results:**

81.03% of parents or legal representatives expressed at least one barrier, the most frequent being availability (49.38%), followed by accessibility (32.24%), acceptability (11.83%) and contact (6.53%).

**Conclusion:**

The majority of parents or legal representatives interviewed reported barriers to accessing health services. Given that these have a negative impact on the health of children with CI, joint interventions are required to reduce them and thus guarantee better health conditions in children with CI and auditory or visual impairment.

Según la Organización Mundial de la Salud (OMS) 1, los defectos congénitos (DC) son alteraciones estructurales o funcionales de órganos o sistemas que ocurren durante la vida intrauterina y se detectan durante el embarazo, el parto o en un momento posterior de la vida; estos se producen a causa de factores genéticos y/o ambientales y generan una importante carga de la enfermedad. En 2015, Sitkin *et al.*^2^ indicaron que los DC representan la asombrosa cifra de 25,3 y 38,8 millones de años de vida ajustados por discapacidad en todo el mundo y ocupan el puesto 17 dentro de las causas de carga de morbilidad.

En Colombia, según datos del Departamento Administrativo Nacional de Estadística (DANE), para el 2020 los DC causaron el 24,70% de las muertes en niños menores de un año 3 y el 1,80% de las muertes fetales 4. Además, Manotas *et al.*^5^ establecieron que la prevalencia general de ciertos DC que alteran la audición o la visión en niños nacidos en Bogotá entre 2002 y 2016 (n=402 657) fue de 24,04 casos por cada 10 000 nacidos vivos.

Los DC del oído son una causa importante de déficit auditivo 6, razón por la cual su análisis ha sido de interés para varios investigadores como Kõsling *et al.*^7^, quienes en 2003 realizaron un estudio en 50 pacientes (43 niños y adultos jóvenes, 7 adultos) con sospecha de malformación del oído interno para comprobar el valor de la resonancia magnética en la identificación de este tipo de defectos y concluyeron que esta prueba puede ser el método de elección en el diagnóstico de este tipo de malformaciones.

Por su parte, las anomalías del desarrollo ocular son responsables de al menos el 25% de los casos de discapacidad visual infantil en el mundo 8, siendo una discapacidad evitable en el 80% de los casos con detección y tratamiento oportuno 9.

Asimismo, se ha descrito que el diagnóstico tardío de los DC y, por tanto, su intervención tardía se ha asociado con una alta morbimortalidad y un alto grado de discapacidad, lo que a su vez representa un importante impacto en los sistemas de salud 10,11.

Por otra parte, se ha establecido que múltiples factores de carácter geográfico, económico y cultural, junto con las condiciones del sistema de salud, son determinantes que pueden generar inequidad en el acceso a los servicios de salud y por ende en los desenlaces en salud 12. Por lo tanto, identificar estas barreras es esencial para generar estrategias de intervención y promover el acceso oportuno y de calidad a los servicios en salud 13,14. En este sentido, en 1978 Tanahashi 15 propuso un modelo de cobertura efectiva en salud basado en la interacción entre la prestación de los servicios y la población. Este modelo plantea cinco etapas que conducen sucesivamente a una intervención de salud deseada. De este modo, las barreras de acceso a la salud se pueden clasificar a partir de cada una de estas etapas, tal como se describe a continuación:

- Disponibilidad: se refiere a la disponibilidad de recursos como mano de obra, instalaciones, medicamentos, entre otros.
- Accesibilidad: hace referencia a que los servicios deben ubicarse dentro de un alcance razonable de las personas que deben beneficiarse de ellos.
- Aceptabilidad: se refiere a la aceptación que tengan las personas de utilizar los servicios.
- Contacto: hace referencia al contacto real entre el proveedor de servicios y el usuario.
- Eficacia: se refiere a la ejecución de un servicio efectivo o una intervención exitosa relacionada con el problema de salud del usuario.

El estudio del impacto de las barreras de acceso a la salud es de gran relevancia ya que estas afectan considerablemente el pronóstico de los pacientes y se presentan en diversos contextos, como se evidencia en la revisión sistemática de Hirmas-Adauy *et al.*^16^, en la cual se encontró que las barreras de acceso de servicios en salud se presentan en países de distinto nivel de desarrollo; en todos los contextos de desarrollo social y económico, y en distintos esquemas de financiamiento y aseguramiento de salud, y que el costo de los medicamentos y la disponibilidad de las consultas médicas y los exámenes constituyen las barreras reportadas con mayor frecuencia.

En Colombia, la Constitución Política de 1991 17 estableció que la salud es un derecho fundamental para todos sus ciudadanos y por tanto la población debe poder acceder a servicios de salud eficaces, oportunos y de calidad para cuidar y promover su salud. Para esto, en 1993, mediante la Ley 100 18, se establecieron políticas y regulaciones sobre las cuales se prestará el servicio público de salud a toda la población colombiana a través del Sistema General de Seguridad Social en Salud (SGSSS). Sin embargo, fue hasta 2015, a partir de la Ley Estatutaria 1751 19, que la salud se concibió como un derecho fundamental y que la afiliación al SGSSS se estableció como de carácter obligatorio e irrenunciable.

Asimismo, de acuerdo con la Ley 1122 del 2007 20, en el sistema de salud colombiano existen dos regímenes de aseguramiento en salud según la capacidad económica de las personas: el régimen contributivo, para quienes pueden realizar un aporte monetario en función de su nivel de ingresos, y el régimen subsidiado para quienes no tienen capacidad de pago. En este punto es importante aclarar que esta ley define el aseguramiento en salud como la administración del riesgo financiero, la gestión del riesgo en salud, la articulación de los servicios que garantice el acceso efectivo y la garantía de la calidad en la prestación de los servicios de salud.

Adicionalmente, en 2015 el Ministerio de Salud y Protección Social emitió la Resolución 1536 21 que establece las disposiciones sobre el proceso de planeación integral para la salud; se planea la afiliación de la población a las Entidades Promotoras de Salud (EPS) y a las Entidades Administradoras de Planes de Beneficio (EAPB), y se establece que estas instituciones tienen entre sus responsabilidades caracterizar toda la población a su cargo en los departamentos, distritos y municipios donde operen.

No obstante, la realidad es que en Colombia el acceso a la salud suele ser complicado, pues como lo reportaron Restrepo-Zea *et al.*^22^, en el país los tiempos de espera para atención en salud son prolongados, la oferta de servicios en salud es baja y la población general desconoce cómo funciona el sistema de salud. Del mismo modo, en un estudio sobre el acceso a la atención en los sistemas de salud de Colombia y Brasil realizado en 2015, García-Subirats 23 encontró que en Colombia existen múltiples barreras, tanto en el acceso como en el uso de servicios de salud, las cuales incluyen problemas en la búsqueda de atención (afiliación, tiempo y dinero) y en la resolución del problema (especialmente en la atención de urgencias), así como determinantes de salud (accesibilidad geográfica, costos de la atención y la exigencia de autorizaciones). De igual forma, en este estudio la autora concluye que existen inequidades a favor de la población con mejor posición socioeconómica en todos los servicios 23.

En atención a los problemas de acceso a la salud de la población con DC, el Instituto de Genética Humana (IGH) de la Pontificia Universidad Javeriana de Bogotá, con el apoyo del Departamento Administrativo de Ciencia, Tecnología e Innovación y la Secretaría de Salud de Bogotá, creó el programa de Atención Integral a Familias con Enfermedades Huérfanas con Compromiso Visual y/o Auditivo (AIVA) para brindar una atención de calidad multidisciplinaria a esta población en Bogotá, Colombia. Para la ejecución del programa AIVA se recibieron datos de 16 hospitales de la ciudad registrados en el Programa de Vigilancia y Seguimiento de Anomalías Congénitas de Bogotá (PVSACB) 24.

Dado el panorama, el objetivo del presente estudio fue identificar las barreras en la atención de la salud en pacientes con DC visuales y auditivos atendidos mediante el programa AIVA.

## MATERIALES Y MÉTODOS

### Tipo y población de estudio

Se realizó un estudio transversal. La población de estudio estuvo constituida por los bebés con DC y posible compromiso auditivo y/o visual nacidos entre el 1.o de agosto de 2016 y el 30 de junio de 2018 y registrados en la base de datos del PVSACB (N=944). Los DC se evaluaron mediante la Clasificación Internacional de Enfermedades CIE-10 25 y se tuvieron en cuenta los recién nacidos clasificados con los siguientes códigos: E50; H02-H22; H26-H50; H69-H75; H90-H91; P35-P37; Q10-Q17 y Q89-Q99. Una vez identificados los casos, se procedió a contactar telefónicamente a sus padres y/o responsables legales, y se logró comunicarse con 596 (63,13%); de estos, 358 (60,06%) negaron alguna alteración auditiva o visual en sus niños y 168 (28,15%) informaron fallecimiento del menor, por lo que finalmente se evaluaron 70 niños, de los cuales en 58 se confirmó el diagnóstico de DC con posible compromiso visual y/o auditivo, siendo esta la muestra de estudio final.

### Procedimientos

En los casos en los que fue posible contactar a los padres y/o responsables legales de los niños, a estos se les explicó el objetivo, las características y el alcance del estudio, y se les preguntó si deseaban participar. A quienes aceptaron se les solicitó llevar a los niños a una valoración médica de ingreso al programa AIVA realizada en el IGH, y en la cual se confirmó el diagnóstico de DC y se formalizó la participación en el estudio mediante la firma del consentimiento informado.

### Variables de estudio

Durante la valoración de ingreso se le realizó una entrevista semiestructurada a los padres y/o responsables legales donde se indagó sobre las barreras de acceso a la salud percibidas por ellos y clasificadas según las primeras cuatro etapas propuestas por Tanahashi 15 en su modelo de cobertura en salud. No se incluyó la quinta etapa que mide la eficacia, debido a que el alcance del programa no permitió evaluar este desenlace. A continuación se describen las cuatro etapas evaluadas.

#### Disponibilidad

Se evaluó la percepción de los padres acerca de las barreras para la afiliación al sistema de salud y la disponibilidad de agendamiento para citas médicas y de cirugías, o para la entrega de medicamentos. En este sentido, las variables de disponibilidad analizadas fueron: sin cita con especialista; sin cita para exámenes; sin cita para control médico; sin autorización de exámenes; sin autorización para cita con especialista; sin autorización de cirugía; sin aseguramiento, definido como la falta de cobertura fuera de la ciudad de nacimiento; sin autorización de cita para control médico, y sin cita para cirugía.

#### Accesibilidad

Debido a que los pacientes requieren con frecuencia asistir a valoraciones médicas, se consideraron tres barreras de acceso: tiempo de espera excesivo (mayor a 30 días) para la asignación de citas; presencia de dificultad para transportarse a las citas (ausencia de transporte particular, escasas rutas públicas y baja frecuencia de tránsito desde el lugar de vivienda), y distancia entre el lugar de residencia y el sitio de atención médica mayor o igual a 10 km (el cálculo de la distancia se realizó mediante el *software* de libre acceso Google Maps).

#### Aceptabilidad

Se evaluó el impacto familiar y social de los DC mediante el instrumento *Impact on Family Scale* (IOFS), el cual fue validado para población colombiana en el año 2010 26. Este resultado se relacionó con tres variables descritas por la metodología de Tanahashi: temor o vergüenza del DC por parte de los dos padres, estigma social por el DC del hijo y desconfianza en la atención del sistema de salud.

#### Contacto

Se valoró mediante la adherencia de los pacientes a los controles médicos y la ausencia injustificada a estos. A los padres y/o representantes legales de los pacientes que abandonaron los controles médicos o el proceso de rehabilitación se les preguntó abiertamente por las causas y los resultados, los cuales se relacionaron con dos variables: sensación de bienestar que lleva a dejar el seguimiento médico y falta de conciencia o conocimiento sobre la enfermedad.

Debido a que los padres y/o representantes legales podían señalar una o más barreras de acceso a los servicios de salud, estas fueron analizadas individualmente para cada uno de los pacientes.

Durante la valoración médica se realizó una entrevista a los padres y/o representantes legales y se revisó la historia clínica de cada paciente con el fin de confirmar las siguientes variables: sexo, edad gestacional, régimen de aseguramiento, EAPB a la cual estaban afiliados, tipo de DC (auditiva, visual, polimalformación y síndromes cromosómicos), lugar de residencia y ubicación del centro de salud al que estaban asignados.

### Análisis estadístico

Los datos se analizaron mediante estadística descriptiva, de la siguiente manera: se calcularon frecuencias absolutas y relativas para las variables cualitativas, y medias y desviaciones estándar o medianas y rangos intercuartílicos, según la distribución de los datos (determinada con la prueba Shapiro-Wilk) para las variables cuantitativas. Los datos se registraron y analizaron en una base de datos creada en el programa Microsoft Excel® 2016.

### Consideraciones éticas

El estudio tuvo en cuenta los principios éticos para las investigaciones médicas en seres humanos establecidos por la Declaración de Helsinki 27 y las disposiciones sobre investigación en salud de la Resolución 8430 de 1993 del Ministerio de Salud de Colombia 28. De igual forma, se contó con la aprobación del Comité de Ética e Investigación de la Facultad de Medicina de la Pontificia Universidad Javeriana según el acta FM-CIE-8612-15 del 27 de agosto del 2015 y se obtuvo el consentimiento informado por parte de los padres y/o representantes legales de los niños después de explicarles los objetivos del estudio, así como los posibles beneficios y perjuicios de participar en este, y garantizarles la confidencialidad de los datos.

## RESULTADOS

La mayoría de los participantes (n=33, 56,90%) fueron de sexo masculino, el DC más frecuente fue el auditivo para ambos sexos y todos los casos tuvieron peso adecuado para la edad gestacional. En la Tabla 1 se presentan las características de las variables analizadas en el estudio.

**Tabla:** 1. Características de la población de estudio (n=58)

Tipo de defecto congénito	Sexo				Peso (g)		Edad gestacional (semanas)	
	Femenino		Masculino					
	Fa	Fr	Fa	Fr	Media	De	Media	De
Visual	2	3,45%	4	6,90%	2697,50	597,61	36,83	3,54
Auditivo	13	22,41%	21	36,21%	3115,00	345,81	38,59	1,16
Sindrómico	10	17,24%	5	8,62%	2520,73	523,17	37,07	2,66
Polimalformación	0	0,00%	3	5,17%	2654,33	1205,11	36,33	3,79
Total	25	43,10%	33	56,90%	2894,29	540,03	37.90	2.20

Fa: frecuencia absoluta; Fr: frecuencia relativa; De: desviación estándar.

En cuanto a las barreras de acceso a los servicios de salud, se encontró que los padres y/o representantes legales de 47 (81,03%) de los 58 pacientes valorados manifestaron al menos una barrera. Al revisarlo por tipo de DC, se encontró que las barreras fueron reportadas todos los padres de niños con DC visuales (n=6), por el 76,47% (n=26) de los padres de niños con DC auditivos, por el 86,66% (n=13) de los padres de niños con DC sindrómicos y por el 66,66% (n=2) de los padres de niños con polimalformación. En cuanto al tipo de régimen de afiliación de salud, se halló que todos los padres de los niños afiliados al régimen subsidiado reportaron barreras y solo el 77,55% de los afiliados al régimen contributivo lo hicieron (Tabla 2).

**Tabla 2:** Frecuencia de barreras de salud según el régimen de afiliación de salud de la población de estudio (n=58)

Tipo de defecto congénito	Presencia de barreras			
	Régimen subsidiado		Régimen contributivo	
	Sí	No	Sí	No
Visual	1 (11,11%)	0 (0,00%)	5 (10,20%)	0 (0,00%)
Auditivo	5 (55,55%)	0 (0,00%)	21 (42,86%)	8 (16,33%)
Sindrómico	2 (22,22%)	0 (0,00%)	11 (22,45%)	2 (4,08%)
Polimalformación	1 (11,11%)	0 (0,00%)	1 (2,04%)	1 (2,04%)
Total	9 (100,00%)	0 (0,00%)	38 (77,55%)	11 (22,45%)

**Figura 1:**
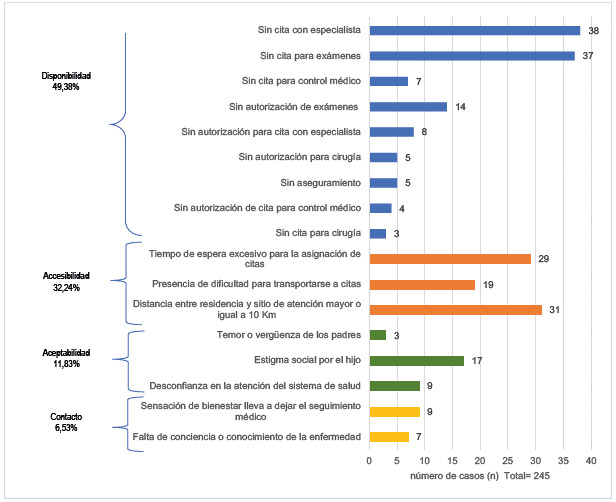
Frecuencia de las barreras de acceso a la salud según el modelo de cobertura de Tanahashi

Las barreras de disponibilidad fueron las más frecuentes (49,38%) (Figura 1), y dentro de estas "sin cita con un especialista" fue la más reportada (38 padres, 29,01%), seguida de "sin cita para exámenes" (37 padres, 28 ,24%( (Tabla 3). Cabe *destacar* que se encontraron 5 _S_asos con barreras de aseguramiento debido a la falta de cobertura fuera de la ciudad de nacimiento.

**Tabla 3:** Distribución de frecuencias de las barreras de disponibilidad según el defecto congénito

Anomalía	Sin cita con especialista	Sin cita para exámenes	Sin cita para control medico	Sin autorización de exámenes	Sin autorización de cita con especialista	Sin autorización para cirugía	Sin aseguramiento	Sin cita para cirugía	Sin autorización de cita para control medico
Microtia	22 (16,79%)	17 (12,97%)	7 (5,34%)	4 (3,05%)	2 (1,53%)	0 (0,00%)	1 (0,76%)	0 (0,00%)	1 (0,76%)
Trisomia21	5 (3,82%)	6 (4,58%)	1 (0,76%)	1 (0,76%)	0 (0,00%)	0 (0,00%)	0 (0,00%)	0 (0,00%)	0 (0,00%)
Polimalformados	2 (1,53%)	2 (1,53%)	2 (1,53%)	2 (1,53%)	1 (0,76%)	0 (0,00%)	2 (1,53%)	0 (0,00%)	0 (0,00%)
Catarata	1 (0,76%)	1 (0,76%)	1 (0,76%)	1 (0,76%)	1 (0,76%)	1 (0,76%)	1 (0,76%)	1 (0,76%)	1 (0,76%)
Coloboma	1 (0,76%)	1 (0,76%)	0 (0,00%)	0 (0,00%)	0 (0,00%)	0 (0,00%)	0 (0,00%)	0 (0,00%)	0 (0,00%)
Glaucoma	3 (2,29%)	3 (2,29%)	2 (1,53%)	3 (2,29%)	1 (0,76%)	3 (2,29%)	1 (0 76%)	1 (0,76%)	2 (1,53%)
Goldenhar	1 (0,76%)	2 (1,53%)	2 (1,53%)	1 (0,76%)	1 (0,76%)	0 (0,00%)	0 (0,00%)	0 (0,00%)	0 (0,00%)
Hiperamonemia	1 (0,76%)	1 (0,76%)	1 (0,76%)	1 (0,76%)	1 (0,76%)	0 (0,00%)	0 (0,00%)	0 (0,00%)	0 (0,00%)
Osteogénesis imperfecta	1 (0,76%)	1 (0,76%)	0 (0,00%)	0 (0,00%)	0 (0,00%)	0 (0,00%)	0 (0,00%)	0 (0,00%)	0 (0,00%)
Treacher Collins	1 (0,76%)	2 (1,53%)	1 (0,76%)	1 (0,76%)	1 (0,76%)	1 (0,76%)	0 (0,00%)	1 (0,76%)	0 (0,00%)
Microftalmia	0 (0,00%)	1 (0,76%)	0 (0,00%)	0 (0,00%)	0 (0,00%)	0 (0,00%)	0 (0,00%)	0 (0,00%)	0 (0,00%)
Total	38 (29,01%)	37 (28,24%)	17 (12,97%)	14 (10,69%)	8 (6,11%)	5 (3,82%)	5 (3,82%)	3 (2,29%)	4 (3,05%)

Clasificación por color de acuerdo con la frecuencia de presentación: 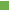 <1% 1-5% 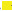 5-10% 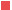 >10%.

Las barreras de accesibilidad fueron las segundas más frecuentes, con 32,24%, siendo "distancia entre residencia y sitio de atención mayor o igual a 10 km" la más reportada (31 padres, 39,24%), seguida de "tiempo de espera excesivo para la asignación de citas" (29 padres, 36,71%). Con respecto a esta última barrera, los padres reportaron más de tres meses de demora para obtener una cita para evaluación médica o para lectura de exámenes de laboratorio (Figura 1).

En tercer lugar se ubicaron las barreras de aceptabilidad, con 11,83%, siendo la desconfianza en el sistema de salud y el temor a la estigmatización social los factores que más influyeron negativamente en la búsqueda y el acceso a los servicios de salud. Finalmente, en cuarto lugar se ubicaron las barreras de contacto, con 6,53%, las cuales estuvieron representadas principalmente por la falta de información y conocimiento sobre la evolución de la enfermedad y la percepción de tener aceptable condición médica, lo que llevó al abandono de los controles y/o tratamientos médicos indicados.

## DISCUSIÓN

En el presente estudio la mayoría de los participantes eran de sexo masculino (56,90%), lo que concuerda con lo encontrado en la literatura; por ejemplo, Tomairek *et al.*^8^, en un estudio transversal realizado entre 2011 y 2014 con 2 500 casos de DC atendidos en un hospital de El Cario, encontraron que 60 participantes sufrieron una o más malformaciones oculares y que la mayoría de estos (61,7%) eran de sexo masculino; de igual forma, varias publicaciones concuerdan en que las malformaciones del oído, como la microtia, tiene una mayor incidencia en hombres 29-31.

En 2014, el comité regional de la Organización Mundial de la Salud OMS 32 para las Américas publicó la Estrategia para el acceso universal a la salud y la cobertura universal de salud, con el objetivo de garantizar que todas las personas tengan acceso a los servicios de salud que necesitan. En el presente estudio se evidenció que la implementación de estas estrategias ha surtido efecto, pues todos los participantes se encontraban afiliados al sistema de salud: el 84,3% al régimen contributivo y el 15,7% al subsidiado. Sin embargo, pese a que todos los participantes tenían cobertura en salud, en el 81,03% (n=47) de los casos los padres y/o representantes legales reportaron barreras de acceso a salud y dentro de estos el 100% (n=9) de los afiliados al régimen subsidiado, lo que evidencia lo reportado por la Organización Panamericana de la Salud (OPS) 33, que indicó que a pesar de que haya progresos en los niveles de cobertura y de acceso a los servicios de salud, esto no garantiza que no hayan barreras de acceso a los servicios de salud.

También es importante mencionar que, a pesar de que según la normativa colombiana la población debe tener acceso equitativo a los servicios de salud, tal como lo reafirman Ruiz-Gómez y Zapata-Jaramillo 34, en el presente estudio se evidenciaron diferencias en la frecuencia de reporte de barreras entre los dos sistemas de afiliación: 100% de los afiliados al régimen subsidiado vs. 77,55% de los afiliados al régimen contributivo, lo cual indica que se deben continuar los esfuerzos para la reducción de las barreras de acceso a salud y a su vez para disminuir las brechas entre los dos sistemas de afiliación.

En el presente estudio se encontró que las barreras de disponibilidad fueron las más frecuentes: se encontró que los padres y/o representantes legales del 65,5% de los niños analizados reportaron baja oferta de profesionales de la salud y el 63,8% indicó falta de recursos estructurales, lo cual limitó el acceso oportuno para citas de atención y para exámenes médicos; esto concuerda con lo descrito por Das *et al.*^35^, quienes analizaron el estado de los servicios de atención oftalmológica en el sudeste asiático durante el año 2015 y encontraron que la disponibilidad de oftalmólogos y de personal auxiliar de oftalmología fue insuficiente, lo cual limitó el acceso y la atención oportuna de las personas.

Las barreras de accesibilidad fueron las segundas más frecuentes, destacándose las dificultades para acceder al transporte y el tiempo excesivo que tardan los niños en llegar al centro de salud asignado para su atención; estas barreras también han sido reportadas con porcentajes significativos en diversos estudios, como el de Das 36, quien llevó a cabo una investigación para establecer el perfil de ceguera y discapacidad visual en el sudeste asiático y hacer una evaluación rápida de la ceguera evitable en esta zona, y encontró que la accesibilidad fue la principal barrera para el uso de los servicios de atención oftalmológica en Maldivas y Timor Leste; el de Mehari *et al.*^37^, quienes encontraron que el 47,9% de los adultos con cataratas en Etiopía reportaron las largas distancias entre el hospital y su hogar como un factor que retrasó la programación de sus cirugías de cataratas; el de Merugumala *et al.*^38^, quienes entrevistaron a 16 madres de niños sordos de India (principalmente de entornos socioeconómicos bajos) y 8 miembros del personal de un centro auditivo benéfico en Hyderabad para identificar las barreras en el manejo oportuno de la sordera y encontraron que las limitaciones económicas y de transporte para llegar al centro de atención fueron las más reportadas, y el de Davey *et* al.^39^, quienes en una revisión sistemática en la que analizaron los determinantes sociales de la salud y los desenlaces en personas con cardiopatías congénitas encontraron asociación entre las barreras de transporte y desenlaces negativos como pérdidas de citas médicas, pérdidas de pacientes durante los seguimientos, mayores reingresos hospitalarios y mayor mortalidad infantil.

Finalmente, las barreras de aceptabilidad y de contacto, que tienen principalmente componentes sociales y culturales, tuvieron una frecuencia de 12,3% y 6,8%, respectivamente. En este punto es importante aclarar que las creencias y el desconocimiento de las enfermedades y los cuidados en salud desempeñan un papel fundamental en la toma de decisiones sobre los pacientes. Al respecto, en Filipinas, Yamaguchi *et al.*^40^ hicieron un estudio transversal sobre los comportamientos de estilo de vida asociados con la progresión y la prevalencia de las enfermedades no transmisibles, y encontraron asociación entre la alta prevalencia de progresión de este tipo de enfermedades y una conciencia insuficiente de los comportamientos que pueden prevenirlas. Otros estudios que han evidenciado que las barreras de accesibilidad y contacto afectan de forma importante a los pacientes son el de Arcoleo *et al.*^41^ -quienes analizaron las experiencias de madres de niños con diagnóstico de asma en México y encontraron, por un lado, que en varios de los casos en los que se suspendía el tratamiento las madres tenían conocimiento limitado sobre la enfermedad y la veían como un cuadro agudo que se presentaba solo cuando el niño tenía los síntomas, por lo que administraban los tratamientos exclusivamente durante estos episodios y como consecuencia no se lograba el adecuado control de la patología, y por el otro, que las madres informaron que los padres, los hermanos y otros miembros de la familia experimentaron sentimientos de miedo, preocupación e impotencia al vivir con un niño con asma- y el de Adogini *et al.*^42^ -quienes analizaron los factores asociados con la detección temprana del cáncer de seno y encontraron que las mujeres que fueron diagnosticadas en estadios más tempranos y que presentaron un menor riesgo de progresar a etapas avanzadas tenían un mayor conocimiento sobre el cáncer de seno y la importancia del autoexamen-.

El presente es uno de los pocos estudios a nivel mundial que analizan el impacto de las barreras de la atención en salud en niños con DC. Sin embargo, en esta investigación no se evaluaron intervenciones enfocadas en la reducción de estas barreras, por lo que se considera pertinente que en el futuro se hagan nuevos estudios que orienten la toma de decisiones para lograr este objetivo.

La mayoría de los padres y/o representantes legales entrevistados reportaron barreras de acceso a los servicios de salud, la cuales se presentaron en los dos sistemas de aseguramiento vigentes en el país. Las barreras de disponibilidad fueron las más frecuentes, seguidas de las de accesibilidad, por lo que se hace necesario un esfuerzo conjunto que involucre a todas las instituciones de salud, especialmente las reguladoras, para mejorar la oportunidad y el acceso a servicios de salud de calidad para todos los pacientes, incluidos los niños con DC con compromiso auditivo y/o visual ♦
